# Applications of artificial intelligence in cancer immunotherapy: a frontier review on enhancing treatment efficacy and safety

**DOI:** 10.3389/fimmu.2025.1676112

**Published:** 2025-10-31

**Authors:** Ji’an Liu, Rao Fu, Yang Su, Zhengrui Li, Xufeng Huang, Qi Wang, Zhengqin Shi, Shouxin Wei

**Affiliations:** ^1^ The Ninth People’s Hospital of Shanghai Jiao Tong University School of Medicine, Shanghai, China; ^2^ Shanghai Xuhui District Dental Center, Shanghai, China; ^3^ Faculty of Dentistry, University of Debrecen, Debrecen, Hungary; ^4^ Department of Oncology, Ruijin Hospital, Shanghai Jiao Tong University School of Medicine, Shanghai, China; ^5^ Department of General Surgery, First Affiliated Hospital of Huzhou University, Huzhou, China; ^6^ Department of Gastrointestinal Surgery, Suining Central Hospital, Suining, China

**Keywords:** artificial intelligence, cancer immunotherapy, immune checkpoint inhibitors, machine learning, predictive modeling, CAR-T cell therapy, biomarkers, safety assessment

## Abstract

Cancer immunotherapy represents a major breakthrough in oncology, particularly with immune checkpoint inhibitors (ICIs) and CAR-T cell therapies. Despite improved outcomes, challenges such as immune-related adverse events (irAEs) and treatment resistance limit clinical use. Artificial intelligence (AI) offers new opportunities to address these barriers, including target identification, efficacy prediction, toxicity monitoring, and personalized treatment design. This review highlights recent advances in AI applications for biomarker discovery, safety evaluation, gene editing, nanotechnology, and microbiome modulation, integrating evidence from clinical and preclinical studies. We also discuss future directions and challenges in applying AI to cancer immunotherapy, aiming to support further research and clinical translation.

## 1 Introduction

The field of cancer treatment has undergone a significant transformation in recent years, with immunotherapy emerging as a revolutionary approach that harnesses the body’s immune system to combat malignancies (1). This innovative treatment modality aims to activate and enhance the immune response against cancer cells, leading to improved patient outcomes and survival rates. The clinical significance of immunotherapy is underscored by its success in various cancer types, including melanoma, lung cancer, and hematological malignancies, where it has demonstrated durable responses and long-term survival benefits (2). However, despite these advancements, the effectiveness of immunotherapy is not universal, and a considerable proportion of patients do not respond adequately (3). The heterogeneity of tumor biology and the immune system’s complexity present significant challenges in optimizing immunotherapeutic strategies. Understanding these dynamics is crucial for the continued development and refinement of cancer immunotherapy as a cornerstone of oncological care (4).

One of the primary challenges facing immunotherapy is the variability in treatment response among patients, often referred to as efficacy heterogeneity. Factors contributing to this variability include the tumor microenvironment, the presence of immune checkpoints, and the individual patient’s immune profile. For instance, while immune checkpoint inhibitors have shown remarkable efficacy in tumors with high mutational burdens, such as melanoma, they have been less effective in “cold” tumors that exhibit low immunogenicity, such as pancreatic cancer. Additionally, immune-related adverse events (irAEs) pose a significant concern, as they can range from mild to severe and may lead to treatment discontinuation. These irAEs arise from the activation of the immune system against normal tissues, complicating the therapeutic landscape and necessitating careful monitoring and management (5, 6).

**Figure 1:**
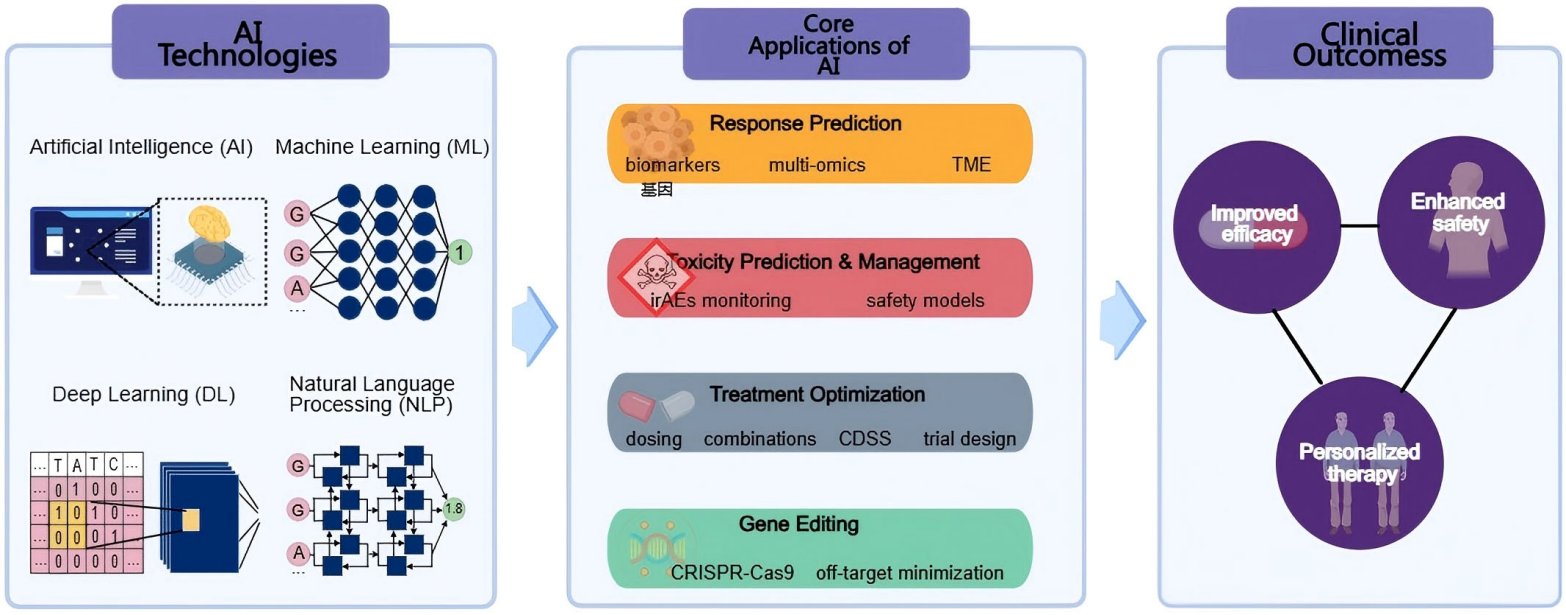
Overview of AI applications in cancer immunotherapy. The schematic illustrates how AI technologies, including Artificial Intelligence (AI), Machine Learning (ML), Deep Learning (DL), and Natural Language Processing (NLP)—support four core application areas: response prediction, toxicity prediction and management, treatment optimization, and gene editing. These applications collectively contribute to improved efficacy, enhanced safety, and personalized therapy in clinical cancer immunotherapy.

The emergence of artificial intelligence (AI) technologies in the healthcare sector offers promising avenues to address these challenges in cancer immunotherapy. AI encompasses computational methods that mimic human decision-making (7). Machine learning (ML), the main branch of AI, allows algorithms to learn from data without explicit programming. ML can be supervised (trained on labeled outcomes, e.g., responders vs. non-responders) or unsupervised (finding patterns in unlabeled data, e.g., tumor subtypes). Deep learning (DL), a subset of ML using neural networks, is especially powerful for complex data such as imaging and genomics. These approaches form the foundation for AI applications in immunotherapy (**Figure 1**). AI has the potential to analyze vast datasets, including genomic, transcriptomic, and clinical information, to identify biomarkers predictive of treatment response. By leveraging machine learning algorithms, AI can assist in the stratification of patients based on their likelihood of benefiting from immunotherapy, ultimately leading to more personalized treatment approaches (8). Furthermore, AI can enhance the design of combination therapies, optimizing treatment regimens that integrate immunotherapy with other modalities, such as chemotherapy or targeted therapies, to improve overall efficacy and minimize adverse effects (9, 10).

This review aims to systematically assess the current applications of AI in enhancing the efficacy and safety of cancer immunotherapy. By focusing on recent advancements and ongoing research, we will explore how AI technologies are being integrated into clinical practice to optimize treatment outcomes for diverse cancer types. The discussion will encompass AI-driven predictive models, the identification of novel therapeutic targets, and the potential for AI to streamline clinical workflows, ultimately paving the way for a more effective and personalized approach to cancer immunotherapy (11, 12). Through this comprehensive evaluation, we seek to highlight the transformative impact of AI on the future of cancer treatment and its role in overcoming the limitations of current immunotherapeutic strategies.

## 2 AI in response prediction

### 2.1 Application of AI in tumor neoantigen recognition

The integration of AI into the identification of tumor neoantigens represents a transformative advancement in cancer immunotherapy. Neoantigens, which are unique to individual tumors due to somatic mutations, hold significant potential for personalized therapeutic strategy. Machine learning approaches rapidly screen and predict tumor-specific neoantigens, improving vaccine design and immunotherapy protocols. These algorithms can analyze vast datasets, including genomic, transcriptomic, and proteomic information, to identify potential neoantigens that may elicit a robust immune response. For instance, AI-driven models can predict the binding affinity of mutated peptides to major histocompatibility complex (MHC) molecules, which is crucial for T-cell recognition and subsequent immune activation (13). This capability reduces the time and resources needed for neoantigen identification, streamlining personalized cancer treatment.

A notable case study exemplifying the application of AI in neoantigen vaccine development is the design of the EVX-01 vaccine, which utilizes the PIONEER™ AI platform. This platform was instrumental in identifying tumor-derived neoantigens for inclusion in the vaccine formulation. In a phase I clinical trial (NCT03715985) enrolling 12 patients with advanced metastatic melanoma, EVX-01 was administered in combination with anti-PD-1 therapy. Patients received six vaccinations (three intraperitoneal followed by three intramuscular) at escalating dose levels (500 µg, 1000 µg, and 2000 µg total peptide), with neoantigens selected by the PIONEER AI platform and formulated with the CAF®09b adjuvant. The vaccine demonstrated a favorable safety profile, with most adverse events limited to grade 1–2 reactions such as injection-site pain, fatigue, and nausea; only two patients experienced grade 3 immune-related events (myositis and nephritis), attributed mainly to anti-PD-1 therapy. Importantly, objective responses were observed in 67% of patients (6 partial responses and 2 complete responses), with durable responses at the highest dose level. EVX-01 induced robust vaccine-specific CD4+ T cell responses in all patients, with CD8+ responses detected in seven patients. Furthermore, the magnitude of T cell responses correlated with peptide dose and PIONEER quality scores, suggesting that the AI platform can effectively prioritize immunogenic epitopes (14). The 48–55 day manufacturing timeline highlights how AI accelerates personalized therapy development. The safety profile observed in clinical trials further underscores the viability of AI-assisted neoantigen identification in enhancing immunogenic responses while minimizing adverse effects.

AI platforms like PIONEER™ are also pivotal in accelerating the development of personalized vaccines by enabling the rapid evaluation of immunogenicity and safety profiles of predicted neoantigens. Simultaneous assessment of multiple neoantigens enables prioritization of those most likely to elicit effective immune responses. This capability is particularly beneficial in the context of tumor heterogeneity, where individual tumors may express unique antigenic profiles that require tailored therapeutic approaches (15). Furthermore, AI’s role in analyzing patient-specific data can lead to the identification of neoantigens that correlate with favorable clinical outcomes, thereby guiding the selection of the most promising candidates for vaccine development.

However, AI-driven neoantigen recognition faces substantial challenges (16). First, data heterogeneity undermines model accuracy: genomic data may be generated via different sequencing platforms with varying error rates, while transcriptomic data often reflects tissue-specific expression patterns that lack standardization across studies. This inconsistency can lead to false-positive predictions of neoantigens. Second, training dataset bias is prevalent—most models are trained on data from Caucasian patients with common cancers (e.g., melanoma), resulting in poor performance when applied to underrepresented ethnic groups or rare tumors.

In conclusion, the application of AI in the recognition and prediction of tumor neoantigens is revolutionizing the landscape of cancer immunotherapy. By streamlining the identification process and enhancing the precision of personalized vaccines, AI technologies are paving the way for more effective and tailored treatment strategies. As ongoing research continues to refine these AI methodologies, the potential for improved patient outcomes in cancer therapy becomes increasingly promising (17). The integration of AI into neoantigen discovery not only exemplifies the convergence of computational science and oncology but also underscores the necessity for continued innovation in the development of personalized cancer treatments (18).

### 2.2 Integration of multidimensional data for predicting immune therapy efficacy

The construction of comprehensive predictive models for the efficacy of immunotherapy in cancer treatment necessitates the integration of various data types, including genomic, transcriptomic, and clinical data. Recent studies have highlighted the significance of combining multi-omics data to enhance predictive accuracy for patient responses to immunotherapy. For instance, the integration of metabolic imaging metrics, such as those obtained from multiparametric PET scans, with genomic and transcriptomic profiles has shown promise in predicting the efficacy of immunotherapies, including PD-1/PD-L1 inhibitors and CAR-T cell therapies (19). This multi-faceted approach allows for a more nuanced understanding of the tumor immune microenvironment (TIME) and its influence on treatment outcomes. Furthermore, machine learning algorithms have been employed to analyze these integrated datasets, enabling the identification of potential biomarkers associated with treatment response. For example, a study demonstrated that specific gene expression patterns correlated with immune cell infiltration and treatment efficacy, thus providing a framework for developing personalized treatment strategies (20).

AI plays a crucial role in enhancing the predictive capabilities of these models. By employing machine learning algorithms, researchers can analyze multi-omics data to uncover hidden patterns that may predict patient responses to immune checkpoint inhibitors and elucidate mechanisms of resistance. A notable application of AI in this context is the development of necroptosis-related gene signatures that have been shown to predict responses to immune checkpoint inhibitors across various cancer types (20). The ability to identify and validate these biomarkers through multi-omics integration not only aids in predicting treatment efficacy but also facilitates the stratification of patients based on their likelihood of benefiting from specific immunotherapies.

Moreover, the identification of biomarkers through machine learning approaches has been instrumental in recognizing potential therapeutic targets that can enhance the effectiveness of immunotherapies. For instance, a recent study utilized integrative multi-omics analysis to classify ovarian cancer patients into molecular subtypes, revealing distinct immune profiles that corresponded with differential responses to immunotherapy (21). This underscores the potential of multi-omics integration in personalizing cancer treatment by tailoring immunotherapy regimens to the unique molecular characteristics of individual tumors.

In addition to genomic and transcriptomic data, the incorporation of clinical data into predictive models is essential for translating these findings into clinical practice. By analyzing clinical outcomes alongside multi-omics data, researchers can develop risk models that account for various patient-specific factors, thereby improving the accuracy of predictions regarding treatment efficacy. For example, a recent study constructed a multi-omics-derived risk score in a large cohort of patients with hepatocellular carcinoma by integrating genomic alterations, transcriptomic expression profiles, and clinical variables. This model effectively stratified patients into high- and low-risk groups with distinct responses to immune checkpoint inhibitor therapy and demonstrated significant predictive value for overall survival (22). Such integrative approaches provide a more comprehensive basis for guiding clinical decision-making and tailoring immunotherapy strategies to individual patients.

Despite these advances, challenges persist. Data integration complexity is a major hurdle: clinical data (e.g., comorbidities, prior treatments) is often stored in unstructured formats (e.g., free-text electronic health records), requiring labor-intensive preprocessing (e.g., NLP-based text extraction) to standardize (23). Additionally, regulatory barriers limit data sharing across institutions due to privacy laws (e.g., HIPAA in the U.S.), reducing the size and diversity of training datasets for AI models (24). This lack of diverse data further exacerbates model bias, as seen in the underperformance of many response-prediction models in elderly or non-Caucasian patients.

Despite these advances, challenges persist. Data integration complexity is a significant barrier: clinical data is often stored in unstructured formats, necessitating labor-intensive preprocessing to standardize it. This lack of diverse data further exacerbates model bias, as seen in the underperformance of many response-prediction models in elderly or non-Caucasian patients (25).

In conclusion, the integration of multidimensional data, including genomic, transcriptomic, and clinical information, is pivotal for constructing robust predictive models of immunotherapy efficacy (26). The application of AI and machine learning algorithms in analyzing these comprehensive datasets holds the potential to revolutionize personalized cancer treatment, enabling clinicians to make informed decisions based on the unique molecular and clinical profiles of their patients. Continued research in this area will undoubtedly enhance our understanding of the complex interplay between the tumor microenvironment and immune responses, ultimately leading to improved outcomes for cancer patients undergoing immunotherapy.

### 2.3 AI-driven optimization of precision immunotherapy strategies

AI technologies have significantly advanced the prediction of immunotherapy outcomes by integrating multi-omics and clinical datasets (27). Machine learning algorithms can identify key biomarkers from genomic, transcriptomic, and proteomic data, enabling more accurate patient stratification and prediction of therapeutic response. For example, integrating tumor microenvironment features with molecular alterations allows AI-driven models to uncover hidden patterns that correlate with treatment efficacy (28). These approaches not only refine patient selection but also provide a deeper understanding of the mechanisms underlying response variability, thereby laying the foundation for more precise immunotherapy strategies.

Taken together, AI-driven approaches to response prediction not only improve patient stratification but also generate insights that can guide subsequent treatment optimization (29). These predictive foundations set the stage for broader applications of AI in tailoring therapeutic strategies, including dosing adjustments, combination regimens, and clinical decision support.

## 3 AI in toxicity prediction and management

### 3.1 prediction and monitoring of IrAEs

The prediction and monitoring of irAEs are crucial components in the management of patients undergoing immune checkpoint inhibitor (ICI) therapy, particularly as these therapies become increasingly prevalent in oncology. AI models have emerged as a significant tool in assessing the risk of severe irAEs, such as cardiac toxicity, which can lead to life-threatening complications. For instance, studies have shown that genetic variations, such as those in the IL7 gene, can predict the likelihood of experiencing irAEs, thereby enabling clinicians to stratify patients based on their risk profiles (30). Additionally, the integration of multimodal data—including biomarkers, imaging studies, and clinical parameters—has been shown to enhance the predictive accuracy of these models. For example, combining genomic data with clinical indicators can provide a more comprehensive risk assessment for patients, allowing for tailored monitoring strategies that align with individual risk factors (31, 32).

The construction of early warning systems utilizing AI-driven algorithms is also gaining traction, with several clinical applications demonstrating their efficacy. These systems leverage real-time patient data to identify early signs of irAEs, facilitating prompt intervention and potentially mitigating severe outcomes. For instance, a predictive model developed using patient-reported outcomes and clinical data has shown promising results in forecasting the onset of irAEs, thereby allowing for timely adjustments in treatment regimens (33). Moreover, the utilization of machine learning techniques to analyze comprehensive datasets has resulted in models that can accurately predict the occurrence of irAEs before they manifest clinically, thus enhancing patient safety and treatment efficacy (34).

In clinical practice, the application of these predictive models has led to improved management of irAEs, with examples including the use of specific biomarkers such as the neutrophil-to-lymphocyte ratio (NLR) and eosinophil counts to forecast adverse events (35). These biomarkers can be integrated into routine clinical assessments, providing oncologists with actionable insights that inform treatment decisions. Furthermore, the development of nomograms based on clinical and laboratory data has been proposed as a method for stratifying patients according to their risk of developing severe irAEs, thereby enhancing the overall management of patients undergoing immunotherapy (36).

Overall, the integration of AI and multimodal data analysis into the prediction and monitoring of irAEs represents a significant advancement in personalized cancer care. By identifying patients at high risk for adverse events, clinicians can implement proactive monitoring strategies, ultimately improving patient outcomes and minimizing the burden of treatment-related toxicities. As research continues to evolve in this area, the potential for AI to transform the landscape of cancer immunotherapy through enhanced safety and efficacy remains promising.

Recent studies have emphasized that predictive biomarkers are pivotal for linking AI models to both treatment response and safety monitoring in immunotherapy. Researchers have highlighted that biomarkers such as PD-L1 expression, tumor mutational burden (TMB), microsatellite instability (MSI), peripheral blood indices including neutrophil-to-lymphocyte ratio (NLR) and lactate dehydrogenase (LDH), circulating tumor DNA, and gut microbiota are not only predictive of ICI efficacy but also associated with immune-related toxicities (37). AI-driven approaches can integrate these multidimensional biomarkers into unified models, capturing complex non-linear associations beyond the capacity of traditional methods. By combining genomic, serological, and microbiome features, machine learning frameworks enable simultaneous identification of likely responders and early detection of patients at high risk for severe irAEs. Furthermore, emerging deep learning strategies that correlate radiomics and digital pathology with biomarker signatures further bridge efficacy prediction and toxicity monitoring, providing clinicians with actionable tools to balance therapeutic benefit and safety in real-world practice. Several studies have evaluated AI-based models for irAE prediction or detection in clinical settings (**Table 1**).

**Table 1:** Representative AI studies on irAE prediction/identification.

Cancer type	Number of pts	Aim of the study	AI method	Conclusion	Limitations	References
Non-small cell lung cancer; melanoma; genitourinary cancers; head and neck cancer	34	Early detection of irAEs through patient-reported outcomes	Machine learning on ePRO data	ML models enabled near-real-time monitoring of symptoms related to irAEs and supported earlier clinical intervention.	Small heterogeneous cohort; limited external validation.	(33)
Esophageal cancer; gastroesophageal junction cancer; gastric cancer; lung cancer	138	Prediction of severe irAEs during PD-1 therapy using blood-based biomarkers	Elastic-net logistic regression	The model identified patients at higher risk of severe irAEs, facilitating proactive management.	Single-center retrospective design; external validation required.	(34)
Lung cancer	74	Prediction of treatment response and irAEs using serum antibody signatures	Resampling-based machine learning	Antibody-based ML models predicted both immunotherapy response and risk of irAEs, showing promise for personalized monitoring.	Small sample size; limited diversity; requires prospective validation.	(35)

Reference numbers in the table correspond to the reference list provided at the end of the manuscript. irAE, immune-related adverse event; ICI, immune checkpoint inhibitor; ePRO, electronic patient-reported outcomes; EHR, electronic health records; CNN, convolutional neural network; NLP, natural language processing; LLM, large language model.

While these studies demonstrate encouraging performance, most are limited by small or single-center cohorts, retrospective design, and a focus on detection rather than true prospective prediction, underscoring the need for multicenter validation and clinically actionable models.

### 3.2 AI-assisted immunotherapy adverse effect management strategies

The integration of AI into the management of adverse effects associated with immunotherapy represents a significant advancement in oncology, particularly in optimizing drug dosing and combination therapy regimens. AI can analyze vast datasets to identify patterns that correlate with adverse reactions, enabling healthcare providers to tailor treatment plans that minimize side effects. For instance, machine learning algorithms can predict the optimal dosages of immunotherapeutic agents based on patient-specific factors, such as genetic profiles and previous treatment responses, thereby reducing the likelihood of adverse events (38). Moreover, AI can assist in the design of combination therapies by evaluating the synergistic effects of various agents, which can lead to improved efficacy while simultaneously lowering the risk of toxicity. This approach allows for a more personalized treatment strategy, ensuring that patients receive the most effective therapies with the least harmful side effects, ultimately enhancing their quality of life during treatment (38, 39).

Machine learning also plays a crucial role in the detection of safety signals associated with immunotherapy. By continuously monitoring patient data, AI systems can identify early warning signs of adverse effects, facilitating prompt intervention. For example, algorithms can analyze electronic health records, laboratory results, and imaging studies to detect changes that may indicate the onset of irAEs such as pneumonitis or colitis (9). This real-time monitoring capability allows clinicians to intervene before these events escalate, improving patient outcomes and potentially reducing hospitalizations related to severe side effects. Furthermore, AI can enhance the predictive accuracy of risk assessments, enabling healthcare providers to stratify patients based on their likelihood of experiencing irAEs. Such individualized risk assessments can guide clinical decisions, such as the need for preemptive treatments or closer monitoring for high-risk patients (17).

In addition to optimizing treatment regimens and enhancing safety signal detection, AI can facilitate personalized risk assessments that promote treatment safety in immunotherapy. By analyzing patient demographics, genetic information, and treatment history, AI systems can provide insights into individual susceptibility to adverse effects. For instance, predictive models can identify patients who may be at higher risk for specific irAEs based on their genetic makeup or pre-existing conditions (40). This personalized approach not only informs clinical decision-making but also empowers patients by providing them with tailored information regarding their treatment plans and potential risks. Consequently, patients can engage in shared decision-making with their healthcare providers, leading to more informed choices about their treatment options (11).

In conclusion, AI-assisted strategies for managing adverse effects in immunotherapy are transforming cancer care by optimizing drug dosing, enhancing safety signal detection, and facilitating personalized risk assessments. These advancements not only improve treatment efficacy but also significantly enhance patient safety and quality of life. As AI technology continues to evolve, its integration into clinical practice will likely lead to more effective and safer immunotherapy regimens, paving the way for a new era in cancer treatment (41).

### 3.3 Clinical data-driven safety model validation and optimization

The integration of large-scale clinical databases into AI model training is pivotal for enhancing the safety and efficacy of cancer immunotherapy. These databases, which encompass diverse patient demographics, treatment regimens, and outcomes, provide a rich resource for training AI algorithms. By harnessing vast amounts of clinical data, AI can identify patterns and correlations that may not be apparent through traditional analytical methods. For instance, AI models can analyze patient responses to various immunotherapies, correlating specific genetic markers or tumor characteristics with treatment outcomes, thereby allowing for more personalized treatment approaches. The ability to leverage real-world evidence from these databases not only aids in the initial training of AI models but also in their continuous refinement as new data becomes available. Furthermore, the validation of AI models against large clinical datasets can enhance their predictive accuracy and generalizability, ensuring that the models are robust and applicable across different patient populations and treatment settings. This approach aligns with the principles of precision medicine, where treatment is tailored to the individual characteristics of each patient, ultimately improving safety and treatment efficacy in cancer care (39, 42).

Cross-validation and methods to enhance model generalization are essential components in the development of AI-driven safety models. Cross-validation techniques, such as k-fold cross-validation, allow researchers to assess the performance of their models by partitioning the dataset into subsets, training the model on some subsets while validating it on others. This process helps to mitigate overfitting, where a model performs well on training data but poorly on unseen data. By ensuring that models are trained and validated on diverse datasets, researchers can better evaluate their predictive capabilities and robustness. Additionally, employing techniques such as data augmentation, regularization, and ensemble learning can further enhance model generalization. For example, ensemble methods, which combine predictions from multiple models, can improve accuracy and reliability by leveraging the strengths of different algorithms. As AI continues to evolve, the incorporation of advanced machine learning techniques, including deep learning and transfer learning, will further bolster the ability of models to adapt to new data and maintain high performance across various clinical scenarios. This iterative process of validation and optimization is crucial for developing AI systems that can effectively monitor patient safety and predict adverse events associated with immunotherapy (12).

Looking ahead, the future trends in AI for safety monitoring in cancer immunotherapy are promising and multifaceted. As AI technologies advance, there is a growing emphasis on developing real-time monitoring systems that can provide immediate feedback on patient responses to treatment. These systems could utilize continuous data streams from wearable devices, electronic health records, and laboratory results to detect adverse reactions or treatment failures early. Moreover, the integration of AI with other emerging technologies, such as genomics and proteomics, can facilitate a more comprehensive understanding of individual patient responses to immunotherapy. This holistic approach enhances prediction of treatment efficacy and aids in identifying potential safety concerns before they escalate. Furthermore, the ethical implications of AI in clinical practice, particularly regarding data privacy and algorithmic bias, will necessitate ongoing dialogue and regulatory oversight to ensure that AI applications are both effective and equitable. As the landscape of cancer treatment evolves, the role of AI in optimizing safety and efficacy will be critical in shaping the future of personalized medicine (43, 44).

## 4 AI in optimizing treatment strategies

### 4.1 AI-assisted personalization of dosing and combinations

The integration of AI into cancer immunotherapy has opened new avenues for the development of personalized treatment regimens tailored to the unique characteristics of individual patients. AI algorithms can analyze vast datasets, including genomic, transcriptomic, and proteomic information, to identify biomarkers that predict responses to immunotherapy (45). For instance, a multi-omics study in hepatocellular carcinoma demonstrated that integrating genomic alterations, transcriptomic profiles, and clinical data enabled accurate patient stratification and prediction of response to immune checkpoint inhibitors (41). Similar approaches in melanoma and non-small cell lung cancer have further validated the predictive value of AI-driven models for immunotherapy efficacy (46). Moreover, AI-driven models can optimize treatment regimens by analyzing historical treatment responses and outcomes, thereby enhancing the efficacy of immunotherapy while minimizing adverse effects. This personalized approach not only improves patient outcomes but also addresses the challenges posed by tumor heterogeneity and the complexity of immune responses.

In addition to response prediction, AI has been increasingly applied to refine dosing and guide combination therapies. By incorporating pharmacogenomic features, drug metabolism data, and immune status, AI models can predict optimal dosing strategies that maximize therapeutic efficacy while minimizing the risk of toxicity. Similarly, machine learning can assess potential drug-drug interactions and synergistic effects, enabling rational design of combination regimens, such as immune checkpoint inhibitors paired with chemotherapy, radiotherapy, or targeted therapies (47). These applications provide a framework for adaptive and patient-specific treatment optimization, representing a shift from a one-size-fits-all model toward precision dosing and rational combination design.

Despite their promise, AI-assisted personalization of dosing and combinations faces practical challenges. Data integration across multi-omics, pharmacological, and clinical sources remains complex, and predictive accuracy often varies across cancer types and patient populations. Furthermore, prospective validation in large-scale clinical trials and regulatory acceptance are necessary to translate these computational insights into routine practice (11). Nevertheless, the convergence of AI with pharmacology and systems biology highlights a future where treatment regimens can be continuously optimized, improving both efficacy and safety in cancer immunotherapy.

### 4.2 Integrating AI with CRISPR-Cas9 for precision gene editing

AI into the CRISPR-Cas9 gene editing landscape has revolutionized the precision and safety of genetic modifications. AI algorithms enhance the accuracy of target recognition by analyzing vast genomic datasets, identifying optimal guide RNA (gRNA) sequences, and predicting off-target effects. This optimization is crucial as it directly correlates with the efficacy of the CRISPR-Cas9 system, especially in therapeutic applications such as CAR-T cell therapy. By leveraging machine learning techniques, researchers can develop predictive models that assess the likelihood of off-target cleavage, thereby minimizing unintended genetic alterations that could lead to adverse effects or reduced therapeutic efficacy. For instance, AI-driven tools like DeepCRISPR and CRISTA have demonstrated significant improvements in identifying gRNAs that not only achieve high on-target editing efficiency but also maintain low off-target activity, thus enhancing the overall safety profile of CRISPR applications in clinical settings (48, 49) (**Table 2**).

**Table 2:** Comparison of selected AI platforms in cancer immunotherapy.

Platform	Application domain	Accuracy	Speed	Clinical validation	Strengths	Limitations
PIONEER™ (Evaxion Biotech)	Neoantigen discovery & personalized vaccine design	High (validated in metastatic melanoma, EVX-01 trial)	Rapid (48–55 days from sequencing to vaccine)	Ongoing clinical trials (EVX-01, EVX-02)	Streamlined neoantigen identification; clinically tested	Limited to vaccine development; requires tumor sequencing
DeepCRISPR	CRISPR-Cas9 target recognition & off-target prediction	High (benchmark outperforming traditional scoring methods)	Fast computational prediction	Preclinical (no direct clinical validation yet)	Reduces off-target risk; improves gRNA design	Lack of clinical trial evidence; dependent on training data quality
CRISTA	CRISPR off-target effect prediction	High (better precision in off-target identification)	Moderate	Preclinical studies	Incorporates sequence + epigenetic features	Computational cost; limited datasets
AI-integrated Radiomics Models	Response prediction, biomarker discovery	Moderate to high (varies by dataset)	Moderate	Retrospective validation in multiple cohorts	Links imaging with immune response	Generalizability concerns; limited prospective validation

Moreover, the reduction of off-target effects is particularly vital in the context of CAR-T cell therapy, where the specificity of engineered T cells against tumor antigens is paramount. AI algorithms can analyze genomic contexts to predict potential off-target sites, allowing for the design of gRNAs that minimize these risks. This capability is essential for ensuring that CAR-T cells effectively target cancer cells without inadvertently attacking healthy tissues, which could lead to severe immunological consequences. The application of AI in refining gRNA design and optimizing the CRISPR-Cas9 editing process not only enhances the therapeutic potential of CAR-T cells but also contributes to a more robust and safer immunotherapeutic approach (49, 50).

A compelling case study illustrating the potential of AI-driven gene editing in CAR-T therapy is the use of AI algorithms to streamline the identification of neoantigens—tumor-specific antigens that arise from mutations in cancer cells. By accurately predicting these neoantigens, AI can facilitate the development of personalized CAR-T cells that are tailored to the unique genetic landscape of a patient’s tumor. This personalized approach significantly improves the chances of a successful immunotherapeutic outcome, as the engineered T cells are more likely to recognize and eliminate cancer cells effectively. The convergence of AI and CRISPR-Cas9 thus paves the way for innovative applications in cancer treatment, potentially expanding the reach of CAR-T therapies beyond hematologic malignancies to solid tumors, which have historically been more challenging to target (49, 51).

In conclusion, the application of AI in optimizing CRISPR-Cas9 target recognition and editing processes represents a transformative advancement in genetic engineering. By enhancing the precision and safety of gene editing, AI not only mitigates the risks associated with off-target effects but also empowers the development of personalized immunotherapies such as CAR-T cell therapy. As research continues to evolve in this interdisciplinary field, the integration of AI and CRISPR technologies holds the promise of revolutionizing cancer treatment and improving patient outcomes across various malignancies (48, 49).

(**Table 2**) provides a comparative overview of representative AI platforms, highlighting differences in accuracy, speed, and clinical validation, which may guide their application in cancer immunotherapy.

### 4.3 AI in quality control and cost optimization in immune cell manufacturing

The integration of AI into the manufacturing processes of immune cells, particularly in the context of cancer immunotherapy, has revolutionized quality control and cost optimization. Automated process optimization plays a crucial role in shortening production cycles, which is vital for ensuring timely treatment delivery. By employing smart sensors and AI-driven algorithms, manufacturers can monitor and adjust key parameters in real-time, effectively responding to variations that may affect cell quality. For instance, recent studies have demonstrated that smart sensors can track and model data generated during the automated cell expansion process, leading to enhanced control over critical performance indicators such as cell quantity and viability (52). This real-time adaptability not only streamlines production but also minimizes the risk of batch failures, ultimately contributing to a more efficient manufacturing process. Furthermore, machine learning techniques assist in predicting cell quality and ensuring batch consistency, thereby reinforcing the reliability of the final product. These advancements in quality assurance are particularly significant given the complex nature of immune cell therapies, which require stringent quality standards to maximize therapeutic efficacy and patient safety.

Machine learning in predicting cell quality has profound implications for cost reduction in CAR-T cell therapy. By improving batch consistency and reducing the likelihood of failed batches, manufacturers can decrease waste and lower production costs, making these therapies more accessible to a broader patient population. The ability to predict and ensure the quality of immune cells not only enhances the therapeutic potential but also addresses the economic barriers associated with advanced immunotherapies. As the healthcare landscape shifts towards personalized medicine, the cost-effectiveness of CAR-T therapies becomes increasingly critical. Research indicates that optimizing the production process through AI can lead to significant cost savings, potentially reducing the overall financial burden on healthcare systems and patients alike (53). This is particularly relevant in the context of CAR-T therapies, where production costs have historically been a barrier to widespread clinical adoption. Using AI technologies, manufacturers can streamline operations, enhance product quality, and ultimately drive down costs, paving the way for broader implementation of these life-saving therapies.

In summary, the incorporation of AI in the immune cell manufacturing process represents a significant advancement in both quality control and cost optimization. Automated process optimization and machine learning-assisted quality predictions are transforming how immune cells are produced, ensuring that high-quality products are delivered efficiently and cost-effectively. As the field of cancer immunotherapy continues to evolve, the role of AI will be paramount in overcoming existing challenges and enhancing the accessibility of these innovative treatments. The future of immune cell therapies will likely hinge on the successful integration of AI technologies, which promise not only to improve patient outcomes but also to make advanced therapies more sustainable and economically viable in the long term.

### 4.4 Future personalized immunotherapy strategies combining gene editing and AI

The integration of multi-omics data guides the design of gene editing strategies in personalized immunotherapy. By leveraging genomic, transcriptomic, and proteomic information, researchers can identify specific mutations and aberrations unique to individual tumors, which can inform the selection of appropriate gene editing targets. For instance, the CRISPR/Cas9 system has been effectively utilized to modify genes associated with immune evasion and tumor growth, allowing for tailored interventions that directly address the molecular underpinnings of a patient’s cancer. The application of multi-omics approaches not only enhances the precision of gene editing but also facilitates the identification of potential biomarkers that can predict treatment responses. This personalized approach is exemplified in studies focusing on head and neck squamous cell carcinoma, where the integration of genetic data has led to the development of targeted therapies that significantly improve patient outcomes (54). Furthermore, the incorporation of epigenetic modifications into gene editing designs can enhance the efficacy of immunotherapeutic strategies by reprogramming the tumor microenvironment to be more conducive to immune attack (55). As such, the future of personalized immunotherapy will likely hinge on the ability to synthesize diverse biological data into actionable insights for gene editing applications.

AI-assisted dynamic treatment adjustments and long-term efficacy monitoring represent another frontier in personalized immunotherapy (**Table 3**). By utilizing machine learning algorithms, clinicians can analyze vast datasets to predict patient responses to specific therapies, enabling adaptive treatment strategies that evolve based on real-time patient data. For example, AI can help identify optimal dosing regimens and timing for gene editing interventions, thereby maximizing therapeutic efficacy while minimizing adverse effects. Additionally, AI-driven predictive models can facilitate the monitoring of long-term treatment outcomes, allowing for timely modifications to therapy based on patient-specific responses. This is particularly relevant in the context of immunotherapy, where the heterogeneity of tumor responses necessitates a flexible and responsive treatment framework. Recent advancements in AI have demonstrated the capability to integrate clinical, genomic, and treatment data to refine patient stratification and improve the precision of immunotherapeutic interventions (9).

**Table 3:** Representative AI applications in cancer immunotherapy.

Application area	AI methods	Clinical relevance	Challenges
Response prediction	ML (random forest, SVM), DL (CNN, RNN)	Stratify responders vs non-responders	Data heterogeneity, bias
Toxicity prediction	ML + multimodal integration	Predict irAEs, stratify risk groups	Limited validation, generalizability
Treatment optimization	ML-based dosing models, reinforcement learning	Optimize dosage and combinations	Regulatory approval, interpretability
Gene editing	DL models (DeepCRISPR, CRISTA)	Improve gRNA design, minimize off-target effects	Off-target uncertainty, ethical issues
Safety monitoring	NLP, real-world evidence mining	Early irAE detection, adaptive monitoring	Privacy, data-sharing barriers

AI, artificial intelligence; ML, machine learning; DL, deep learning; SVM, support vector machine; CNN, convolutional neural network; RNN, recurrent neural network; irAEs, immune-related adverse events; gRNA, guide RNA; NLP, natural language processing; EHR, electronic health records.

Cross-disciplinary collaborative innovations are essential for advancing the development of precise immunotherapy. The intersection of computational biology, genomics, and clinical research fosters an environment where novel therapeutic strategies can emerge. For instance, partnerships between bioinformaticians, oncologists, and geneticists can accelerate the identification of actionable targets for gene editing while simultaneously developing AI models that predict treatment outcomes. Collaborative efforts in research have already yielded promising results, such as the development of CAR-T cell therapies enhanced by CRISPR technology, which demonstrate improved efficacy against various malignancies (50). Furthermore, interdisciplinary approaches that combine insights from immunology, bioengineering, and nanotechnology are leading to the creation of innovative delivery systems for gene editing tools, enhancing their precision and reducing off-target effects (56). As the field of personalized immunotherapy continues to evolve, fostering cross-disciplinary collaborations will be crucial in overcoming current challenges and unlocking the full potential of gene editing and AI in cancer treatment (**Table 3**).

## 5 Discussion

The integration of AI technologies into cancer immunotherapy represents a transformative advancement in the field of oncology. This review has highlighted the significant potential of AI across various critical aspects of immunotherapy, including efficacy prediction, safety assessment, gene editing optimization, nanotechnology carrier design, and gut microbiome regulation. The multifaceted role of AI not only enhances the precision of treatment plans but also fosters the development of personalized therapeutic strategies that are tailored to the unique genetic and phenotypic profiles of individual patients (57).

AI synthesis of multidimensional data advances personalized medicine by identifying biomarkers predictive of response and adverse events. This capability is particularly crucial in immunotherapy, where the balance between therapeutic efficacy and the risk of immune-related adverse events is delicate (58). By leveraging AI algorithms to analyze large datasets from clinical trials and real-world evidence, researchers are increasingly able to refine treatment protocols, thereby improving clinical outcomes while minimizing potential risks (59).

At the same time, these opportunities are tempered by significant challenges. Many current AI models are trained on genomic and clinical datasets disproportionately derived from patients of European ancestry, limiting generalizability to more diverse populations and risking further inequities in healthcare. Reproducibility also remains problematic, as algorithms often depend on institution-specific preprocessing pipelines and lack standardized external validation, resulting in inconsistent performance across clinical settings. In addition, regulatory barriers—including concerns about data privacy, model interpretability, and the absence of clear approval pathways—continue to slow clinical translation. As we stand on the cusp of a new era in cancer treatment, addressing these limitations is essential. Robust frameworks are needed to ensure that AI applications can accommodate the complexity of cancer biology and the heterogeneity of patient responses, while safeguarding patient rights and promoting equitable access to innovative therapies. However, as we stand on the cusp of a new era in cancer treatment, it is essential to acknowledge the challenges that accompany the adoption of AI-driven approaches (60). Issues surrounding data privacy, the generalizability of models across diverse populations, and the ethical implications of AI in healthcare must be addressed to ensure the responsible deployment of these technologies. The complexity of cancer biology and the heterogeneous nature of patient responses necessitate robust frameworks that can accommodate the nuances of AI applications while safeguarding patient rights and ensuring equitable access to innovative therapies.

Looking ahead, the future of AI in cancer immunotherapy will likely be characterized by increased interdisciplinary collaboration and the integration of multi-omics data (61). By harnessing insights from genomics, proteomics, metabolomics, and microbiomics, researchers can develop a more comprehensive understanding of tumor biology and the immune response. This holistic approach will not only enhance the precision of immunotherapy but also facilitate the identification of novel therapeutic targets and strategies (62). However, realizing this potential will also depend on overcoming several practical barriers. Future progress requires access to large and diverse datasets, yet existing resources are often fragmented and biased toward certain populations, limiting model generalizability. The integration of sensitive genomic and clinical data also raises privacy and security concerns, highlighting the need for clear governance frameworks and transparent patient consent. Furthermore, effective implementation will depend on specialized personnel, including clinicians trained in digital health and data scientists capable of translating complex algorithms into actionable insights. Addressing these challenges through data-sharing collaborations, privacy-preserving computational techniques, and workforce training will be essential to ensure that AI can deliver on its promise in cancer immunotherapy (63).

In summary, AI in cancer immunotherapy is an ongoing process that holds immense promise for the future of oncology. As we continue to navigate the complexities of this evolving landscape, it is imperative that we foster collaboration among clinicians, researchers, data scientists, and ethicists. Together, we can address the existing challenges and leverage the full potential of AI to achieve a dual enhancement of treatment efficacy and safety. This collaborative effort will be instrumental in advancing the field of precision tumor immunotherapy and ultimately improving outcomes for patients battling cancer.
